# The EuroMyositis registry: an international collaborative tool to facilitate myositis research

**DOI:** 10.1136/annrheumdis-2017-211868

**Published:** 2017-08-30

**Authors:** James B Lilleker, Jiri Vencovsky, Guochun Wang, Lucy R Wedderburn, Louise Pyndt Diederichsen, Jens Schmidt, Paula Oakley, Olivier Benveniste, Maria Giovanna Danieli, Katalin Danko, Nguyen Thi Phuong Thuy, Monica Vazquez-Del Mercado, Helena Andersson, Boel De Paepe, Jan L deBleecker, Britta Maurer, Liza J McCann, Nicolo Pipitone, Neil McHugh, Zoe E Betteridge, Paul New, Robert G Cooper, William E Ollier, Janine A Lamb, Niels Steen Krogh, Ingrid E Lundberg, Hector Chinoy

**Affiliations:** 1 Division of Musculoskeletal and Dermatological Sciences, Centre for Musculoskeletal Research, School of Biological Sciences, Faculty of Biology, Medicine and Health, Manchester Academic Health Science Centre, The University of Manchester, Manchester, UK; 2 Greater Manchester Neurosciences Centre, Salford Royal NHS Foundation Trust, Salford, UK; 3 Institute of Rheumatology, Prague, Czech Republic; 4 Department of Rheumatology, China-Japan Friendship Hospital, Beijing, China; 5 University College London GOS Institute of Child Health and NIHR GOSH Biomedical Research Centre, Great Ormond Street Hospital for Children NHS Trust, London, UK; 6 Department of Rheumatology, Odense University Hospital, Odense, Denmark; 7 Department of Neurology, University Medical Center Göttingen, Göttingen, Germany; 8 Myositis UK, Southampton, UK; 9 Département de Médecine Interne et Immunologie Clinique, Hôpital Pitié-Salpêtrière, AP-HP, UPMC, Paris, France; 10 Clinica Medica, Dipartimento di Scienze Cliniche e Molecolari, Università Politecnica delle Marche & Ospedali Riuniti, Ancona, Italy; 11 Division of Immunology, University of Debrecen, Debrecen, Hungary; 12 Department of Rheumatology, Bach Mai Hospital, Bach Mai Hospital, Hanoi Medical University, Hanoi, Viet Nam; 13 División de Medicina Interna, Universidad de Guadalajara, Guadalajara, Jalisco, Mexico; 14 Department of Rheumatology, Oslo University Hospital, Oslo, Norway; 15 Department of Neurology, Ghent University Hospital, Ghent, Belgium; 16 Department of Rheumatology, University Hospital Zurich, Zurich, Switzerland; 17 Department of Rheumatology, Alder Hey Children’s NHS Foundation Trust, Liverpool, UK; 18 Department of Rheumatology, Arcispedale S. Maria Nuova-IRCCS of Reggio Emilia, Reggio Emilia, Italy; 19 Royal National Hospital for Rheumatic Diseases, Bath, Bath and North East Somer, UK; 20 Department of Pharmacy and Pharmacology, University of Bath, Bath, UK; 21 MRC/ARUK Institute of Ageing and Chronic Disease, University of Liverpool, Liverpool, UK; 22 Division of Population Health, Health Services Research and Primary Care, School of Health Sciences, Faculty of Biology, Medicine and Health, Manchester Academic Health Science Centre, The University of Manchester, Manchester, UK; 23 Zitelab Aps, Copenhagen, Denmark; 24 Unit of Rheumatology, Department of Medicine, Karolinska University Hospital, Solna, Karolinska Institutet, Stockholm, Sweden; 25 Department of Rheumatology, Salford Royal NHS Foundation Trust, Manchester Academic Health Science Centre, Salford, UK; 26 The National Institute for Health Research Manchester Musculoskeletal Biomedical Research Unit, Central Manchester University Hospitals NHS Foundation Trust, Manchester Academic Health Science Centre, The University of Manchester, Manchester, UK

**Keywords:** Disease registries, Myositis

## Abstract

**Aims:**

The EuroMyositis Registry facilitates collaboration across the idiopathic inflammatory myopathy (IIM) research community. This inaugural report examines pooled Registry data.

**Methods:**

Cross-sectional analysis of IIM cases from 11 countries was performed. Associations between clinical subtypes, extramuscular involvement, environmental exposures and medications were investigated.

**Results:**

Of 3067 IIM cases, 69% were female. The most common IIM subtype was dermatomyositis (DM) (31%). Smoking was more frequent in connective tissue disease overlap cases (45%, OR 1.44, 95% CI 1.09 to 1.90, p=0.012). Smoking was associated with interstitial lung disease (ILD) (OR 1.32, 95% CI 1.06 to 1.65, p=0.013), dysphagia (OR 1.43, 95% CI 1.16 to 1.77, p=0.001), malignancy ever (OR 1.78, 95% CI 1.36 to 2.33, p<0.001) and cardiac involvement (OR 2.40, 95% CI 1.60 to 3.60, p<0.001).

Dysphagia occurred in 39% and cardiac involvement in 9%; either occurrence was associated with higher Health Assessment Questionnaire (HAQ) scores (adjusted OR 1.79, 95% CI 1.43 to 2.23, p<0.001). HAQ scores were also higher in inclusion body myositis cases (adjusted OR 3.85, 95% CI 2.52 to 5.90, p<0.001). Malignancy (ever) occurred in 13%, most commonly in DM (20%, OR 2.06, 95% CI 1.65 to 2.57, p<0.001).

ILD occurred in 30%, most frequently in antisynthetase syndrome (71%, OR 10.7, 95% CI 8.6 to 13.4, p<0.001). Rash characteristics differed between adult-onset and juvenile-onset DM cases (‘V’ sign: 56% DM vs 16% juvenile-DM, OR 0.16, 95% CI 0.07 to 0.36, p<0.001). Glucocorticoids were used in 98% of cases, methotrexate in 71% and azathioprine in 51%.

**Conclusion:**

This large multicentre cohort demonstrates the importance of extramuscular involvement in patients with IIM, its association with smoking and its influence on disease severity. Our findings emphasise that IIM is a multisystem inflammatory disease and will help inform prognosis and clinical management of patients.

## Introduction

The idiopathic inflammatory myopathies (IIM), or ‘myositis spectrum disorders’, are a rare and heterogeneous group of multisystem autoimmune diseases. IIM traditionally encompasses polymyositis (PM), dermatomyositis (DM) and juvenile dermatomyositis (JDM). Inclusion body myositis (IBM) has become recognised as an entity distinct from PM, identified by a characteristic distribution of muscle weakness and treatment-resistant course. Additionally, skeletal muscle inflammation can occur in the context of other connective tissue diseases (CTDs), termed CTD-overlap myositis. Immune-mediated necrotising myopathy (IMNM) and the antisynthetase syndrome (ASS) are also now recognised as distinct entities under the IIM umbrella.^1^ The importance of serotype in predicting clinical features and prognosis is becoming increasingly recognised.^2^

Estimates of the prevalence of IIM vary widely from 0.55 to 17.50 per 1 00 000 people.^3^ The rarity and heterogeneity of IIM has hampered research efforts and impeded the delivery of large-scale interventional clinical trials.^4^ Consequently, the therapeutic evidence-base in IIM is remarkably limited.^5 6^ Recently, several IIM research databases and registries have been created to pool resources and expertise, facilitating completion of several international IIM research studies.^7–11^

We describe the data held within EuroMyositis, the largest IIM disease registry, highlighting the differing clinical characteristics of each IIM diagnostic subtype and analyse associations with extramuscular involvement, malignancy, environmental exposures and disease severity.

## Methods

### The EuroMyositis Registry

Several smaller registries were integrated in 2003 to produce the EuroMyositis Registry (https://euromyositis.eu/) (see online supplementary appendix A).

10.1136/annrheumdis-2017-211868.supp1 Supplementary Appendix 1

Anonymised downloads from those agreeing to participate (Belgium, China, Czech Republic, Hungary, Italy, Mexico, Norway, Sweden, Switzerland, the UK and Vietnam) were obtained on 15 August 2016, including 92% (3487/3790) of all cases in the Registry. During data processing, 420 cases were excluded from further analysis; for 317 cases, confirmation of diagnosis was not available, for 55 cases, conflicting data entries were identified and the remaining 48 cases had rare IIM subtypes (see online supplementary appendix B). A total of 3067 cases (81% of the whole Registry) were included in the final analysis.

Analysed sections of the Registry included demographics, clinical features and environmental/lifestyle exposures, including smoking and toxins (asbestos, silica, fibreglass, solvents and coal dust). Where available (64%, 1951/3067), the autoantibody profile for each case was obtained to facilitate accurate diagnostic subtype classification. Specific analysis of the associations between serotype and phenotype will be the subject of a separate publication. Disease severity assessments made using the International Myositis Assessment & Clinical Studies Group Core Set Measures ‘disease activity’ and ‘disease damage’ toolkits^12^ were obtained where available, as were records of any medications prescribed for IIM treatment. Longitudinal data were available for some cases (19%, 596/3067), although we limited this report to cross-sectional analysis using information from the last recorded patient visit.

### Definitions

Investigators at each site initially determined the diagnostic subtype for each case according to the criteria employed by the Registry (see online supplementary appendix B). In addition, rather than retain traditional subtype designation of cases as either PM, DM or JDM, and given the growing consensus that the different IIM subtypes, particularly ASS and IMNM have distinct clinical, pathological and serological characteristics,^13–16^ we performed a process of retrospective subtype reclassification using the data available in the Registry at the time of study.

PM, DM and JDM cases met Bohan and Peter ‘definite’ or ‘probable’ diagnostic criteria.^17 18^ The Registry specifies that these criteria should only be applied (and thus permit inclusion of the patient in the Registry as a PM, DM or JDM case), if known infectious, toxic, metabolic, dystrophic or endocrine myopathies have been excluded by appropriate evaluations and that no exclusion criteria are met (see online supplementary appendix B). IBM cases met either the Medical Research Council, Griggs *et al* or European Neuromuscular Centre diagnostic criteria.^19–21^

Those with suspected PM, DM or JDM, who did not fulfil these criteria were classified as ‘unspecified myositis’ and excluded from further analysis, unless they met the criteria for ASS (in which case they were analysed within the ASS group, see below) or had myositis overlapping with a CTD (in which case they were analysed with the CTD-overlap myositis group). CTD-overlap myositis was defined as PM, DM, JDM or unspecified myositis coexisting with a CTD that met relevant diagnostic criteria.^22–26^ This report uses the term JDM for current adults with juvenile-onset (<18 years) disease. Six cases of ‘juvenile PM’ were excluded from further analysis due to the rarity of this diagnostic entity and the difficulty in drawing conclusions from such a small sample.

For the purposes of this report, we pooled those with ASS (including where felt to be occurring in association with another IIM diagnostic subtype) into a single category by applying criteria proposed by Connors *et al*.^27^ This included retrospective reclassification of those with PM, DM, JDM or CTD-overlap myositis cases as having ASS if they possessed an antisynthetase autoantibody (anti-Jo1, anti-PL-7, anti-PL-12, anti-EJ, anti-OJ, anti-KS or anti-Zo autoantibodies), where results of these were available. These were combined with cases where the recruiting clinician deemed that criteria for ASS were met at the time of recruitment (see online supplementary appendix B). We also reclassified those with ‘unspecified myositis’ and amyopathic DM^28^ as ASS if they possessed an antisynthetase antibody *and* had coexisting interstitial lung disease (ILD), Raynaud’s phenomenon, arthritis or mechanics’ hands. Remaining cases with clinically amyopathic DM were analysed as part of the DM group (n=38). We excluded a small number of cases where conflicting data were identified. This included 28 patients with PM, IMNM and IBM that had the presence of a DM-specific rash recorded and 27 patients with DM where the presence of a DM-specific rash could not be confirmed.

The Registry categorises statin-related myotoxicity (SRM) cases using definitions suggested by Alfirevic *et al* (SRM1–6).^29^ We reclassified as IMNM any case with statin-associated IMNM (SRM6) or SRM occurring in association with 3-hydroxy-3-methylglutaryl-coenzyme A reductase (anti-HMGCR) autoantibodies. Remaining cases with SRM1-5 were excluded from further analysis (n=16). We also reclassified as IMNM any case of PM that had anti-SRP or anti-HMGCR autoantibodies. All diagnostic subtype classifications were applied in a mutually exclusive manner (ie, cases could not be assigned to two IIM subtype groups). A summary of all diagnostic reclassifications is shown in online supplementary appendix B.

Malignancy is recorded in the EuroMyositis Registry regardless of the relationship to the diagnosis of IIM. We assigned cases a label of ‘cancer-associated myositis’ (CAM), where malignancy was diagnosed within 3 years of IIM diagnosis.^30 31^ Smoking was defined as having ever smoked at least one cigarette per day for more than 1 year. Cardiac involvement was defined as the occurrence of pericarditis, myocarditis, arrhythmia or sinus tachycardia occurring due to the IIM disease process. ILD was defined by chest X-ray or CT, and abnormal pulmonary function tests, and occurring as part of the IIM disease process. Disease onset was the date of onset of the first symptoms of IIM. Environmental toxin exposure refers to prior exposure to any of asbestos, silica, fibreglass, solvents or coal dust. Further details of the definitions used by the EuroMyositis Registry are contained in online supplementary appendix A.

### Statistics

Downloaded data were imported into STATA for Windows V.13.0 (College Station, Texas, USA) for processing. Cross-sectional descriptive statistical analysis was performed. For continuous variables, normally distributed data were summarised by calculation of means and SD. Non-normally distributed data were summarised using medians and IQR. Associations were assessed using logistic regression and expressed as OR and 95% CI. This was performed unadjusted, except with regard to analysis of disease activity data from the last patient visit, which was adjusted for disease duration. Kaplan-Meier analysis and proportional hazards regression was used to analyse differences in the interval between disease onset and diagnosis. In this case, the HR is presented to indicate the likelihood of a diagnosis being made over time. Where frequencies are presented, the denominator may vary between different variables because of missing data. No imputation was performed and only complete cases for each variable were analysed. A p value of <0.05 was considered as statistically significant.

## Results

### Case characteristics

Data regarding 3067 cases from 11 countries were analysed. The most common diagnoses were DM (31%, 949/3067), PM (27%, 813/3067) and ASS (17%, 512/3067) (table 1). Of those with CTD-overlap myositis, systemic sclerosis (SSc) was the most common coexisting CTD (39%, 141/358). Most cases were Caucasian (80%, 2155/2681), and 69% (2058/3002) were female. Those with IBM were more likely to be male than those with other diagnostic subtypes (61% (142/233) IBM vs 29% (802/2769) for remainder, OR 3.83, 95% CI 2.91 to 5.04, p<0.001).

**Table 1:** Demographic and clinical information of cases in the EuroMyositis Registry

	Dermatomyositis	Polymyositis	Antisynthetase syndrome	Connective tissue disease-overlap myositis	Inclusion body myositis	Immune- mediated necrotising myopathy	Juvenile dermato-myositis	Total n (%)
Number of cases—n (% of total)	949 (31)	813 (27)	512 (17)	358 (12)*	240 (8)	105 (3)	90 (3)	3067 (100)
Gender—% male | % female (n=3002)	30 | 70	29 | 71	31 | 69	21 | 79	61 | 39	36 | 64	32 | 68	944 (32) male 2058 (69) female
Ethnicity—% per diagnosis (n=2681)								
Caucasian	75	74	86	86	96	93	76	2155 (80)
Asian/Oriental	15	20	6	10	3	4	12	332 (12)
Hispanic	7	4	1	1	0	2	9	98 (4)
Black African	3	3	5	3	1	1	3	81 (3)
Other	0	0	2	1	0	0	0	15 (1)
Mean age in years at disease onset (SD) (n=2427)	49 (15)	50 (15)	48 (15)	45 (15)	61 (10)	56 (15)	10 (5)	49 years (SD 16)
Mean age in years at diagnosis (SD) (n=2000)	51 (15)	52 (15)	49 (15)	48 (15)	65 (10)	57 (15)	10 (5)	51 years (SD 17)
Median interval in months between disease onset and diagnosis (IQR) (n=1668†)	5 (2–11)	8 (3–19)	7 (3–13)	11 (4–24)	41 (24–72)	7 (4–12)	8 (3–22)	8 months (IQR 3–22)
**Clinical features—% per diagnosis**								
Myopathic muscle weakness (n=2521)	92	98	90	94	92	94	91	2352 (93)
Rash‡ (n=1993)	100	0	44	32	0	0	100	1077 (54)
Raynaud’s phenomenon (n=1903)	25	28	51	60	8	20	18	643 (34)
Periungal erythema (n=1305)	52	6	32	33	2	15	37	434 (33)
Arthritis (n=2288)	20	20	50	42	8	10	23	632 (28)
Mechanics’ hands (n=1958)	22	8	38	16	1	4	7	363 (19)
Calcinosis (n=1314)	7	1	3	10	1	0	44	78 (6)
Ulceration (n=1152)	13	2	3	10	0	2	5	79 (7)

Disease onset is defined as the date of onset of the first symptoms of idiopathic inflammatory myopathy.

*Associated connective tissue diseases: systemic sclerosis (39%, 141/358), Sjögrens syndrome (15%, 54/358), mixed connective tissue disease (15%, 52/358), rheumatoid arthritis (9%, 32/358), systemic lupus erythematosus (9%, 32/358), other (13%, 47/358).

†Excludes 281 cases where diagnosis and onset are recorded with the same date.

‡Includes Gottron’s papules/sign heliotrope, rash, ‘V’ sign, shawl sign and erythroderma.

The mean age at IIM diagnosis was 51 years (SD 17) (table 1). IBM cases (mean age at diagnosis 65 years, SD 10) and IMNM cases (mean age at diagnosis 57 years, SD 15) were older at time of diagnosis when compared with the remainder of the adult-onset myositis cohort (IBM: p<0.001; IMNM: p=0.003). The overall median interval between disease onset and IIM diagnosis was 8 months (IQR 3–22, n=1668). This was significantly longer for IBM cases (median 41 months (IQR 24–72), HR 0.38, 95% CI 0.33 to 0.44, p<0.001) and significantly shorter for DM, ASS and IMNM cases (DM: median 5 months (IQR 2–11), HR 1.71, 95% CI 1.54 to 1.90, p<0.001; ASS: median 7 months (IQR 3–13), HR 1.28, 95% CI 1.13 to 1.44, p<0.001; IMNM: median 7 (IQR 4–12), HR 1.28, 95% CI 1.02 to 1.61, p=0.037) when compared with the remainder of the cohort.

Heliotrope rash and Gottron’s papules/sign were observed in similar proportions in those with DM and JDM. However, the shawl and ‘V’ signs were less common in those with JDM (shawl sign: 45% (234/522) of DM vs 15% (6/40) of JDM, OR 0.22, 95% CI 0.09 to 0.53, p=0.001; ‘V’ sign: 56% (308/554) of DM vs 16% (7/43) of JDM, OR 0.16, 95% CI 0.07 to 0.36, p<0.001). Calcinosis occurred in 6% (78/1314) of cases overall but was more common in those with JDM (44% (23/52) of JDM vs 4% (55/1262), OR 17.4, 95% CI 9.45 to 32.04, p<0.001) and CTD-overlap myositis (10% (16/165) of CTD-overlap vs 5% (62/1149), OR 1.88, 95% CI 1.06 to 3.35, p=0.031) when compared with the remainder of the cohort. Of the ASS cases, 90% (439/487) had myopathic muscle weakness, 71% (357/502) ILD, 51% (198/385) Raynaud’s phenomenon, 50% (238/472) arthritis and 38% (146/380) mechanics’ hands.

### Environmental exposures

Overall, 37% (611/1646) of cases were smokers (table 2). Smoking was more common in CTD-overlap myositis cases compared with the remainder of the cohort (45% (103/231) vs 36% (508/1415), OR 1.44, 95% CI 1.08 to 1.90, p=0.012). Prior exposure to environmental toxins was observed more frequently in those with IBM (28% (21/75) vs 16% (136/855), OR 2.06, 95% CI 1.20 to 3.52, p=0.008). Environmental toxin exposure was also more common in smokers, compared with those that had never smoked (26% (71/273) vs 13% (73/571), OR 2.40, 95% CI 1.66 to 3.46, p<0.001).

**Table 2:** Environmental exposures, extramuscular complications and disease severity assessments of cases in the EuroMyositis Registry

	Dermatomyositis	Polymyositis	Antisynthetase syndrome	Connective tissue disease-overlap myositis	Inclusion body myositis	Immune- mediated necrotising myopathy	Juvenile dermatomyositis	Total n (%)
**Environmental exposures—% per diagnosis**								
Current or previous smoker (n=1646)	33	39	42	45	35	29	20	611(37)
Environmental toxin exposure (n=930)	16	17	21	15	28	4	0	157 (17)
**Extramuscular complications— % per diagnosis**								
Interstitial lung disease (n=2442)	21	17	71	32	3	10	6	720 (30)
Cardiac involvement (n=1715)	9	9	11	12	4	10	7	156 (9)
Malignancy ever (n=2788) Of these—% with each type*:	20	8	12	13	16	7	0	374 (13)
*Breast*	22	23	17	16	8	14	–	*19%*
*Bowel*	8	7	12	14	21	0	–	*10%*
*Ovarian*	13	2	2	3	0	0	–	*7%*
*Lung*	10	7	14	9	0	0	–	*9%*
*Other*	52	63	61	59	72	86	–	*58%*
‘Cancer -associated myositis’† (n=2701)	9	3	3	3	5	3	–	132 (5)
Dysphagia (n=1945)	43	35	26	53	50	36	16	767 (39)
**Disease severity assessments at last visit— median (IQR), *n***								**Median (IQR), *n***
Duration of follow-up in years‡	2.6 (1.5–6.1), *186*	2.8 (1.3–5.6), *124*	3.2 (1.8–6.5), *123*	3.8 (2.1–6.6), *91*	3.3 (1.5–5.9), *33*	2.2 (0.7–5.6), *23*	1.3 (0.5–7.1), *16*	3.0 (1.5–6.1), *596*
MMT-8 score (0–80)	75 (64–79), *156*	72 (61–78), *89*	75 (69–80), *93*	71 (59–77), *55*	63 (53–70), *21*	69 (57–77), *21*	78 (70–80), *7*	73 (63–79), *442*
*Physician-completed* global disease activity VAS (0–100)	10 (1–26), *258*	10 (2–26), *156*	9 (1–24), *163*	8 (1–27), *107*	14 (4–29), *39*	16 (10–53), *36*	10 (0–23), *13*	10 (1–26), *772*
*Patient-completed* global disease activity VAS (0–100)	30 (5–55), *203*	45 (28–55), *103*	40 (10–55), *123*	47 (22–64), *83*	44 (24–67), *32*	42 (10–60), *30*	3 (1–16), *13*	40 (11–57), *587*
HAQ-DI (0–3)	0.50 (0–1.25), *239*	0.88 (0.25–1.50), *132*	0.63 (0–1.25), *142*	0.88 (0.38–1.50), *98*	1.82 (1.38–2.50), *38*	0.56 (0.13–2.13), *26*	0 (0–0.13), *17*	0.75 (0.13–1.50), *692*
Creatine kinase (as ratio of ULN)	0.44 (0.29–0.97), 6*3*	1.12 (0.45–3.20), *62*	0.63 (0.35–2.06), *40*	0.66 (0.31–1.90), *19*	1.79 (1.17–2.02), *5*	1.28 (0.63–2.79), *8*	0.56 (0.39–1.85), *11*	0.63 (0.37–1.78), *208*
Extramuscular disease activity VAS (0–100)	7 (0–22), *237*	4 (0–15), *141*	8 (0–18), *149*	6 (0–16), *100*	0 (0–15), *35*	0 (0–15), *29*	12 (0–27), *11*	5 (0–18), *702*
Myositis Damage Index global VAS (0–100)	16 (3–34), *146*	20 (5–38), *84*	19 (8–34), *85*	26 (8–36), *58*	47 (39–62), *22*	14 (3–40), *18*	29 (11–41), *8*	20 (6–38), *421*

*Multiple malignancies were recorded in some cases, therefore total may exceed 100%.

†Malignancy diagnosed within 3 years of the idiopathic inflammatory myopathy diagnosis.

‡For cases with >1 visit with any disease severity assessment recorded. Environmental toxin exposure includes exposure to asbestos, silica, fibreglass, solvents or coal dust.

HAQ-DI, Health Assessment Questionnaire-Disability Index; VAS, visual analogue scale; MMT-8, manual muscle test-8 score; ULN, upper limit of normal.

### Disease activity and disease damage assessments

At the last patient visit, lower manual muscle testing-8 (MMT-8) scores (adjusted OR 0.95, 95% CI 0.93 to 0.98, p<0.001) and higher Health Assessment Questionnaire-Disability Index (HAQ-DI) scores (adjusted OR 3.85, 95% CI 2.52 to 5.90, p<0.001) were observed in those with IBM compared with the remainder of the cohort (table 2). IBM cases also had a higher Myositis Damage Index (MDI) global VAS compared with the remainder of the cohort (median 47 (IQR 39–62) vs 19 (IQR 5–36), adjusted OR 1.05, 95% CI 1.03 to 1.07, p<0.001).

Cases with ASS had a higher MMT-8 score at the last patient visit compared with the remainder of the cohort (adjusted OR 1.03, 95% CI 1.01 to 1.05, p=0.019). Cases with DM, JDM and ASS had lower HAQ-DI scores compared with the remainder of the cohort (DM: adjusted OR 0.74, 95% CI 0.60 to 0.91, p=0.004. JDM: adjusted OR 0.09, 95% CI 0.02 to 0.47, p=0.004. ASS: adjusted OR 0.72, 95% CI 0.57 to 0.93, p=0.011).

### Extramuscular involvement and malignancy

#### Interstitial lung disease

Overall, 30% (720/2442) of cases had ILD (table 2), observed most frequently in those with ASS (71% (357/502) vs 19% in remainder of cohort (363/1940), OR 10.7, 95% CI 8.6 to 13.4, p<0.001). ILD was least frequent in those with IBM (3%, 7/218) and JDM (6%, 4/72). In cases with ILD, current or previous smoking and prior exposure to environmental toxins were observed more frequently than in those without ILD (smoking: 41% (191/462) vs 35% (387/1114), OR 1.32, 95% CI 1.06 to 1.65, p=0.013; toxin exposure: 22% (57/256) vs 14% (93/651), OR 1.72, 95% CI 1.19 to 2.48, p=0.004) (table 3). At their last visit, cases with ILD had a higher extramuscular disease activity VAS (median 10 (IQR 2–22) vs 3 (IQR 0–15), adjusted OR 1.02, 95% CI 1.01 to 1.03, p=0.001) and MDI global VAS (median 25 (IQR 13–40) vs 14 (IQR 4–37), adjusted OR 1.01, 95% CI 1.00 to 1.02, p=0.005) (table 3).

**Table 3:** Clinical characteristics in those with interstitial lung disease, cardiac involvement, malignancy and dysphagia within the EuroMyositis Registry

	Interstitial lung disease 720/2442 (30%)			Cardiac involvement 156/1715 (9%)			Malignancy 374/2788 (13%)			Dysphagia 767/1945 (39%)			Total n (%)
	*Present*	*Absent*	*p Value*	*Present*	*Absent*	*p Value*	*Present*	*Absent*	*p Value*	*Present*	*Absent*	**p Value**	
Mean age at IIM disease onset in years (SD)	49 (15)	49 (17)	0.903	50 (14)	49 (17)	0.602	57 (14)	48 (16)	**<0.001**	51 (15)	48 (17)	**<0.001**	49 years (SD 16)
Gender (% male)	31	33	0.387	37	33	0.436	33	32	0.740	36	33	0.173	944/3002 (32)
Current or previous smoking (%)	41	35	**0.013**	55	34	**<0.001**	51	37	**<0.001**	42	34	**0.001**	611/1646 (37)
Environmental toxin exposure (%)	22	14	**0.004**	27	14	**0.006**	20	18	0.634	20	15	**0.060**	157/930 (17)
Median number of recorded medications used to treat myositis (IQR)	2 (2–4)	2 (1–3)	**<0.001**	3 (2–4)	2 (1–3)	**0.016**	2 (1–3)	2 (2–3)	0.123	2 (2–3)	2 (1–3)	**0.042**	two medications (IQR 1–3)
**Disease severity assessments at last visit— median (IQR)**													**Median (IQR), *n***
MMT-8 score (0–80)	73 (66-78)	74 (63-79)	0.780	67 (61-77)	73 (62-78)	0.200	70 (61-78)	74 (63-79)	0.377	71 (59-78)	73 (64-78)	**0.044**	73 (63-79), *442*
*Physician-completed* global disease activity VAS (0–100)	11 (3-25)	9 (0–24)	0.119	11 (5-26)	11 (2–28)	0.468	7 (1-25)	10 (2-26)	0.469	10 (2-28)	10 (2-25)	0.135	10 (1-26), *772*
*Patient-completed* global disease activity VAS (0–100)	42 (14-57)	38 (10-60)	0.299	50 (31-66)	41 (12-59)	**0.007**	47 (24-64)	38 (10-55)	**0.009**	46 (17-65)	37 (10-53)	**0.007**	40 (11-57), *587*
HAQ-DI (0–3)	0.75 (0.13–1.38)	0.70 (0.13–1.37)	0.272	1.00 (0.38–1.63)	0.67 (0.13–1.38)	**0.012**	1.13 (0.63–1.63)	0.63 (0.13–1.38)	**<0.001**	1.00 (0.25–1.63)	0.63 (0.13–1.13)	**<0.001**	0.75 (0.13–1.50), *692*
Creatine kinase (as ratio of ULN)	0.45 (0.33–1.18)	0.67 (0.41–2.00)	0.542	0.87 (0.66–2.19)	0.66 (0.40–2.05)	0.712	0.40 (0.23–0.58)	0.66 (0.39–1.85)	0.32	0.63 (0.34–1.32)	0.71 (0.39–2.21)	0.259	0.63 (0.37–1.78), *208*
Extramuscular disease activity VAS (0–100)	10 (2-22)	3 (0–15)	**0.001**	12 (3-25)	7 (0–19)	**0.017**	5 (0–19)	6 (0–18)	0.548	7 (0–21)	5 (0–16)	**0.003**	5 (0–18), *702*
Myositis Damage Index global VAS (0–100)	25 (13-40)	14 (4-37)	**0.005**	31 (12-48)	20 (5–38)	**0.047**	37 (22-53)	17 (5-35)	**<0.001**	25 (10-43)	17 (3-32)	**0.001**	20 (6-38), *421*

Environmental toxin exposure includes exposure to asbestos, silica, fibreglass, solvents or coal dust. p Values are derived from a logistic regression model that includes adjustment for time since disease onset where data from the last patient visit are analysed. Values in bold signify p<0.05.

HAQ-DI, Health Assessment Questionnaire-Disability Index; VAS, visual analogue scale; MMT-8, manual muscle test-8 score; ULN, upper limit of normal.

#### Cardiac involvement

Nine per cent (156/1715) of cases had cardiac involvement (table 2). This was most frequently observed in those with CTD-overlap myositis (12%, 27/230). Overall, those with SSc were more likely to have cardiac involvement compared with those without SSc (18% (13/74) vs 9% (143/1641), OR 2.23, 95% CI 1.20 to 4.16, p=0.011). Cardiac involvement was less frequent in those with IBM compared with the remainder of the cohort (4% (8/185) vs 10% (148/1530), OR 0.42, 95% CI 0.20 to 0.87, p=0.020). Smoking was significantly more frequent in those with cardiac involvement compared with those without (55% (56/102) vs 34% (426/1265), OR 2.40, 95% CI 1.60 to 3.60, p<0.001) (table 3). Cardiac involvement was also associated with exposure to environmental toxins (27% (18/68) vs 14% (97/702), OR 2.25, 95% CI 1.26 to 4.01, p=0.006).

At the last patient visit, the presence of cardiac involvement was associated with a higher patient-completed global disease activity VAS (median 50 (IQR 31–66) vs 41 (12-59), adjusted OR 1.02, 95% CI 1.00 to 1.03, p=0.007), HAQ-DI (median 1.00 (IQR 0.38–1.63) vs 0.67 (IQR 0.13–1.38), adjusted OR 1.52, 95% CI 1.10 to 2.12, p=0.012), extramuscular disease activity VAS (median 12 (3-25) vs 7 (0–19), adjusted OR 1.02, 95% CI 1.00 to 1.03, p=0.017) and MDI global VAS (median 31 (IQR 12–48) vs 20 (IQR 5–38), adjusted OR 1.01, 95% CI 1.00 to 1.03, p=0.047) (table 3).

#### Gastrointestinal involvement

Dysphagia was observed in 39% (767/1945) of cases and was more common in those with CTD-overlap myositis (53% (137/258), OR 1.90, 95% CI 1.46 to 2.47, p<0.001) (table 2). The presence of dysphagia was more frequent in those with SSc (67% (63/94), OR 3.31, 95% CI 2.13 to 5.14, p<0.001). The rate of dysphagia was also higher in those with IBM compared with the remainder of the cohort (50% (111/224), OR 1.60, 95% CI 1.21 to 2.11, p=0.001).

Smoking was more frequent in those with dysphagia (42% (247/584) vs 34% in those without dysphagia (322/951), OR 1.43, 95% CI 1.16 to 1.77, p=0.001) (table 3). At the last patient visit, dysphagia was associated with a higher patient-completed global disease activity VAS (median 46 (IQR 17–65) vs 37 (IQR 10–53), adjusted OR 1.01, 95% CI 1.00 to 1.02, p=0.007), HAQ-DI (median 1.0 (IQR 0.25–1.63) vs 0.63 (IQR 0.13–1.13), adjusted OR 1.60, 95% CI 1.30 to 1.96, p<0.001), extramuscular global VAS (median 7 (IQR 0–21) vs 5 (IQR 0–16), adjusted OR 1.02, 95% CI 1.01 to 1.03, p=0.003) and MDI global VAS (median 25 (IQR 10–43) vs 17 (IQR 3–32), adjusted OR 1.02, 95% CI 1.01 to 1.03, p=0.001). The MMT-8 score was lower in those with dysphagia (median 71 (IQR 59–78) vs 73 (IQR 64–78), adjusted OR 0.98, 95% CI 0.96 to 0.99, p=0.044).

#### Malignancy

**Figure 1:**
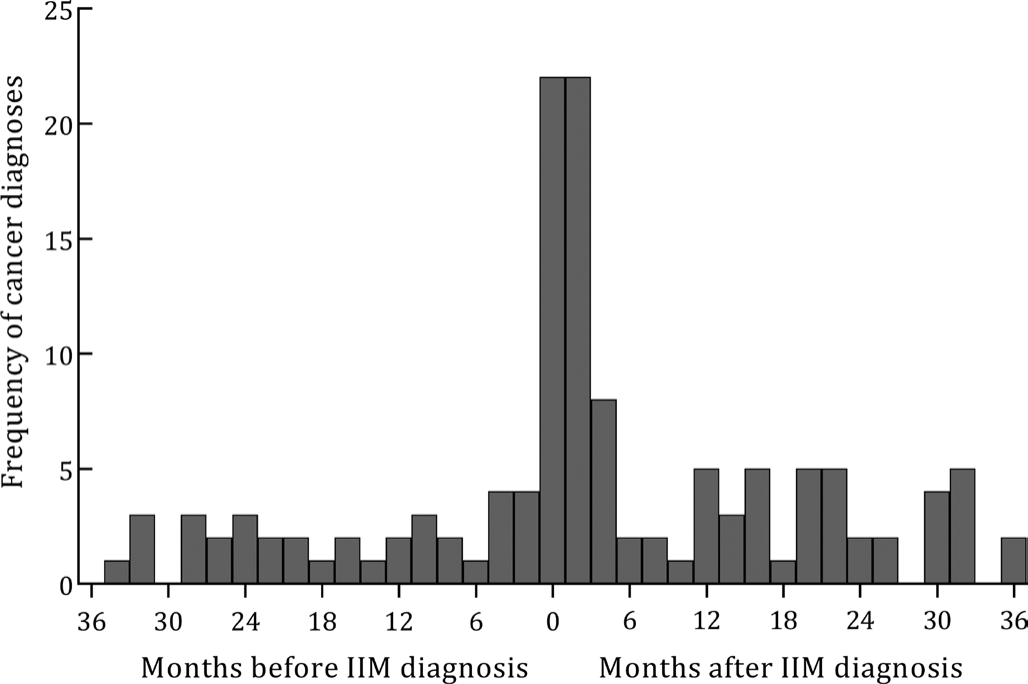
Onset of malignancy in relation to date of diagnosis of idiopathic inflammatory myopathy (IIM) for cases with cancer-associated myositis. Month 0 indicates date of IIM diagnosis.

Malignancy occurred in 13% (374/2788) of cases (table 2). Breast cancer was the most frequently observed cancer subtype (affecting 19% of those with cancer (70/374)). DM cases had a higher frequency of malignancy (20% (166/841) vs 11% (208/1947) in non-DM cases, OR 2.06, 95% CI 1.65 to 2.57, p<0.001). No malignancy was recorded in cases with JDM. Of those with cancer, CAM was defined in 46% (132/287; onset dates missing for remaining 87). CAM was more common in those with DM compared with the remainder of the cohort (9% (72/795) vs 3% (60/1906), OR 3.06, 95% CI 2.01 to 4.36, p<0.001). In those with CAM, the median interval between IIM diagnosis and cancer diagnosis was 1 month (ie, cancer onset 1 month after IIM diagnosis, IQR −3 to +12 months) (figure 1).

Smoking was more frequent in those with malignancy (51% (133/263) vs 37% (472/1294), OR 1.78, 95% CI 1.36 to 2.33, p<0.001) (table 3). Those with malignancy were older at the time of IIM disease onset, compared with those without malignancy (57 (SD 14) vs 48 (SD 16) years, OR 1.05, 95% CI 1.04 to 1.05, p<0.001). The presence of malignancy was associated with a higher patient-completed global disease activity VAS (median 47 (IQR 24–64) vs 38 (IQR 10–55), adjusted OR 1.01, 95% CI 1.00 to 1.02, p=0.009), HAQ-DI (median 1.13 (IQR 0.63–1.63) vs 0.63 (IQR 0.13–1.38), adjusted OR 1.60, 95% CI 1.26 to 2.04, p<0.001) and MDI global VAS (median 37 (IQR 22–53) vs 17 (IQR 5–35), adjusted OR 1.03, 95% CI 1.02 to 1.05, p<0.001) (table 3).

### Medication

A total of 2613 instances of usage of medications to treat IIM were recorded in 1023 cases (table 4). Oral glucocorticoid usage was recorded in 98% (969/993) of cases. Higher proportions of intravenous immunoglobulin usage were evident for cases with JDM (25%, 12/49), IMNM (18%, 9/50) and IBM (17%, 8/47). Cases with ASS had the highest proportions with recorded usage of both cyclophosphamide (39%, 44/114) and rituximab (14%, 26/184).

**Table 4:** Medications used to treat idiopathic inflammatory myopathy in the EuroMyositis Registry

% within each diagnostic subtype with recorded use of each medication	Dermatomyositis (n=353)	Polymyositis (n=206)	Antisynthetase syndrome **(n=184)**	Connective tissue disease-overlap myositis (n=134)	Inclusion body myositis **(n=47)**	Immune- mediated necrotising myositis (n=50)	Juvenile dermatomyositis (n=49)	**Overall total** n (% per medication)* (2613 medications in 1023 cases)
Glucocorticoids	98	100	98	96	89	98	96	969/993 (98)
*Disease modifying antirheumatic drugs*								
Methotrexate	69	76	60	72	82	82	74	500/704 (71)
Azathioprine	44	52	60	51	35	59	57	311/615 (51)
Ciclosporin	31	27	31	23	4	28	40	152/545 (28)
Antimalarials	37	11	16	29	7	0	48	131/533 (25)
Mycophenolate	17	20	31	29	24	13	33	119/511 (23)
Tacrolimus	1	5	2	0	0	0	0	9/487 (2)
Leflunomide	0	3	3	4	0	6	6	11/489 (2)
*Biological therapies*								
Rituximab	6	4	14	7	2	12	0	72/1025 (7)
Other biologic†	7	4	11	20	48	19	22	62/493 (13)
*Immunomodulatory therapies*								
IVIg	11	13	7	7	17	18	25	118/1025 (12)
Plasma exchange	2	2	0	0	0	0	0	5/490 (1)
*Other*								
Cyclophosphamide	15	14	39	18	4	17	6	101/520 (19)
Other (not specified)	5	3	8	10	14	0	11	34/501 (7)
*Topical therapies*								
Topical glucocorticoids	7	2	2	1	4	0	6	18/489 (4)
Topical tacrolimus	0	0	0	1	0	0	0	1/485 (0)
Median number of recorded medications per case (IQR)	2 (1–3)	2 (1–3)	3 (2–4)	2 (2–3)	2 (2–3)	2 (1–3)	2 (1–3)	two medications (IQR 1–3)

*Some cases have received mediation from multiple categories.

†Includes antitumour necrosis factors, anakinra and abatacept.

IMNM, immune-mediated necrotising myopathy; IVIg, intravenous immunoglobulin.

When considering all forms of IIM, the preferred steroid sparing agents were methotrexate (71%, 500/704) and azathioprine (51%, 311/615). In those with ASS, usage rates of azathioprine and methotrexate were similar (60% (77/128) and 60% (76/127), respectively). A high proportion of IBM cases had medication usage recorded in the ‘other biologic’ category (48%, 14/29) relating to participation of Swedish patients with IBM in a clinical trial of anakinra.^32^

## Discussion

Using the large EuroMyositis Registry dataset, we have identified several important new associations, including the strong influence of extramuscular involvement (malignancy, cardiac involvement and dysphagia) on disease severity. This was demonstrated by higher disease activity and damage scores, including worse functional performance according to the HAQ-DI. We found that smoking and environmental toxin exposure was associated with the occurrence of extramuscular involvement (ILD, cardiac involvement, malignancy and dysphagia), although this observation may relate more to direct toxic effects rather than be related to the IIM disease process itself.

We have also used data from the Registry to confirm several previously described observations. This includes the link between malignancy and DM, the different demographic characteristics of those with IBM compared with other IIM diagnostic subtypes and the differing skin disease characteristics of DM and JDM. We also found that cases with CTD-overlap disease, especially those with SSc, were at increased risk of cardiac involvement and dysphagia. We have demonstrated a similar frequency of ILD^27^ and cardiac involvement^33^ in IIM as described in other sources in the literature. The frequency of malignancy we identified was towards the lower range of that reported.^1^

The literature described complex associations between environmental exposures and IIM. Smoking has been shown to interact with serotype (particularly anti-Jo1 autoantibody status) and genotype in IIM, and this may explain some of our findings.^34^ Additionally, several other environmental factors have been investigated, particularly ultraviolet light exposure, seasonal birth patterns and prior infections.^35–37^ Thus, while our observational data cannot imply a causative role for smoking or environmental toxin exposure in the development of extramuscular manifestations of IIM, it is possible that these environmental factors could be of pathogenic relevance.

Disease registries are increasingly facilitating large-scale observational studies in IIM. In the USA, the MYOVISION registry has recently reported on factors associated with a reduced health-related quality of life in IIM.^38^ Factors identified included a diagnosis of IBM and the presence of ILD. We demonstrated a significantly higher HAQ-DI score in those with IBM compared with those with other IIM diagnostic subtypes. However, we did not demonstrate similar findings with regard to the presence of ILD. This may be explained by the fact that our analysis was restricted to understanding differences between IIM subtypes, whereas the MYOVISION authors compared data for IIM cases against normative data from the general population and from rheumatoid arthritis cases.

Our analysis has several limitations, many of which are inherent to analysis of disease registries. We did not perform any data validation, including verification of diagnosis. Differing local practices, for example, local methods of detecting cardiac involvement or malignancy, may have influenced the way data were recorded at individual sites and the definition used in the registry for some features (eg, ILD) may not include use of gold standard diagnostic techniques. Most had only cross-sectional data recorded, meaning that the incidence of some clinical features might be underestimated. In other cases, reporting bias may mean that some reported frequencies are overestimates. Usage rates of certain sections of the Registry also varied between centres, making analysis difficult in some cases. Additionally, in some cases it is possible that the associations demonstrated might be influenced by confounders that we have not accounted for.

Data now comprising the Registry were first recorded as early as 1999 in some cases. At that time, IMNM had not been recognised as a specific IIM subtype, there were no available classification criteria for ASS and several antisynthetase antibodies were yet to be discovered. Despite our attempts to minimise inclusion of misdiagnosed cases, there remains the possibility that some cases, particularly those with PM, could have been misdiagnosed. We also highlight the fact that several alternative proposed diagnostic and classification criteria for IIM are available, use of which may have influenced the results. The complexity of IIM and the lack of consensus diagnostic criteria or definitions of each subtype remain a significant problem for patients, clinicians and researchers alike. Such issues are likely to improve after ratification of the forthcoming European League Against Rheumatism/American College of Rheumatology IIM classification criteria.^39^

Further recruitment into the EuroMyositis Registry will increase our power to detect rarer associations and further elucidate rarer disease subtypes such as juvenile-PM. The steering committee welcomes applications for implementation in additional paediatric and adult centres (https://euromyositis.eu).
