# Supplementary material for: The EuroMyositis registry: an international collaborative tool to facilitate myositis research
Source: Ann Rheum Dis. 2017 Aug 30;77(1):30–9. doi: 10.1136/annrheumdis-2017-211868 (PMC5754739; doi:10.1136/annrheumdis-2017-211868)
Supplement: Supplementary Appendix 1 [file annrheumdis-2017-211868supp001.pdf]

The EuroMyositis Registry: An International Collaborative Tool to Facilitate Myositis Research

Supplementary Appendices:

Supplementary Appendix A 2

The EuroMyositis Registry 2

Background 2

Current capabilities 5

Outputs 5

Future developments 6

Supplementary Appendix B 9

EuroMyositis Registry Definitions 9

Summary of Case Reclassification 13

References 15

Supplementary Appendix A

The EuroMyositis Registry

Background

EuroMyositis (https://euromyositis.eu/) is the largest IIM disease registry. Three centres took the initiative to create the Registry: The University of Manchester (Manchester, UK), Rheumatology Institute (Prague, Czech Republic) and Karolinska Institutet (Stockholm, Sweden). In the UK, it evolved from data captured as part of the Adult Onset Myositis Immunogenetic Collaboration (AOMIC). This was set up in 1999 and later became the UK Myositis Network (UKMYONET). In Sweden, a similar project called SweMyoNet was established in 2003 and in the Czech Republic, the myositis section of National Registry of Inflammatory Rheumatic Diseases, formed in 2002, was used as the basis for data entry in to EuroMyositis. Records from these separate datasets were entered in to the EuroMyositis Registry in 2003, using the criteria from Bohan and Peter as inclusion criteria.

A Delphi process and consensus discussion among Rheumatology and Neurology experts from these centres led to creation of uniform data collection *pro-forma*. The EuroMyositis Registry also incorporates the International Myositis Assessment & Clinical Studies Group (IMACS) disease activity and disease damage core set measures [1,2].

To facilitate data input, storage and access, a web-based interface was created using the Plone platform (an open source Python-based content management system) with extensions shared with the DANBIO-IT-platform (https://danbio-online.dk). All data are encrypted and stored on secure and automatically backed-up servers approved by authorities in Denmark. The Registry can be accessed from any computer with internet access (Supplementary Appendix Figure 1).

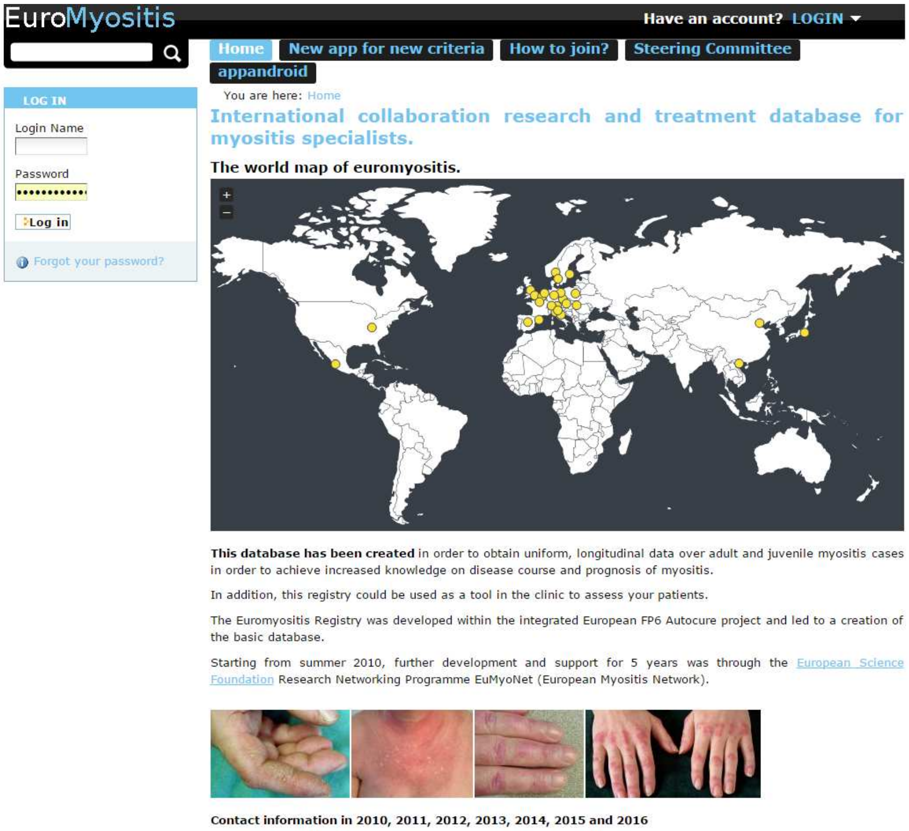

__Supplementary Appendix Figure 1:__ The homepage of the EuroMyositis registry website (https://euromyositis.eu/)

The EuroMyositis registry now includes 20 centres in 16 countries (correct as of 25/08/2016). A summary of the country of origin of patients included in our study is shown in Supplementary Table 1 and Supplementary Appendix Figure 2. Several other centres are currently involved in the application and set-up process. Ethical approvals are required at each centre where the registry is implemented and informed consent is obtained from all included patients. The exact structures used to collect data vary from country to country. For example, in the UK, the project is coordinated from the University of Manchester, but 63 individual centres contribute data.

Each centre may only access their individual dataset by default. This can be done on an individual patient basis using the website, often useful during clinical consultations, or by downloading a file containing the whole dataset in a spreadsheet format. Most data are input manually, but there are facilities for uploads of data to the registry *en-masse* and the ability to synchronise with other systems, including electronic health records.

Several centres outside Europe are now participating, increasing ethnic variety within the dataset which initially consisted principally of European Caucasians. In addition, the Registry

has been recently expanded to include a section relating specifically to JDM, although this data was not analysed as part of this study.

The EuroMyositis administrative structure includes a steering committee and affiliated external IT-expert. The steering committee meets 3-4 times a year to discuss ongoing development of the registry, consideration of requests for use in new centres and consideration of formal requests to use the data in specific research studies, for which specific ethical approval from recruited patients has been obtained.

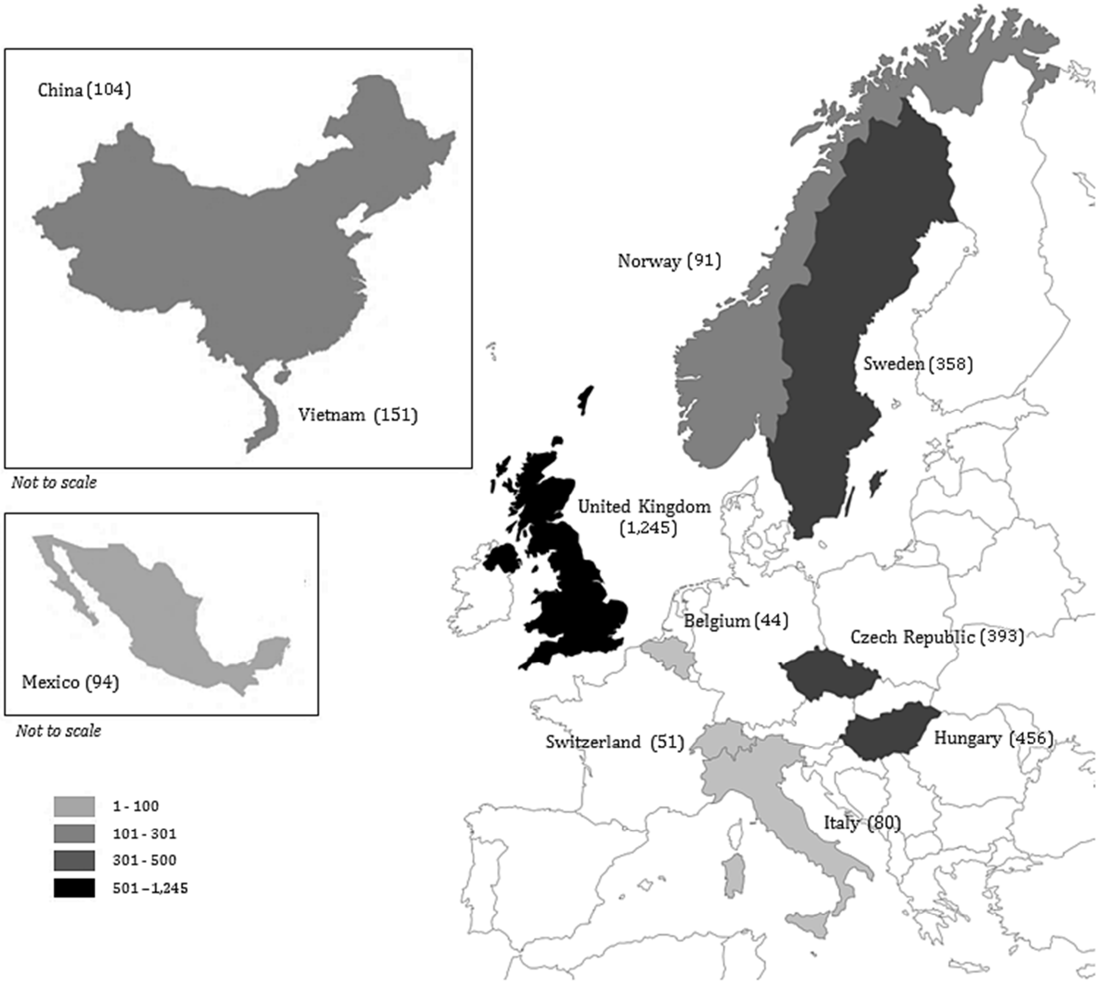

Supplementary Appendix Figure 2: Map indicating case contributions to the EuroMyositis Registry per country included in this study. Depth of shading indicates the total number of patients contributed from each country (see key).

Current capabilities

Within the Registry, each patient record is arranged into two main sections. Firstly, a 'core' dataset includes diagnostic details, associated investigation results, demographic information and baseline clinical characteristics. Autoantibody results and muscle biopsy findings can be included. Secondly, 'per visit' data can then be added for each patient (see Supplementary Appendix Table 1 for per country contributions to this section of the Registry). This includes clinical outcome measures as well as medication prescriptions, which can be updated longitudinally. Data added to this section also populates a patient 'scoreboard' that summarises clinical outcome measures over time (Supplementary Appendix Figure 3) and can be used in the clinical setting to obtain a snapshot of disease activity and help facilitate discussions with patients about their progress.

Scoreboard

PMDM_[redacted]GBMAN[redacted]

Dermatomyositis

	A 10-12-2009	B 25-03-2010	C 10-06-2010	D 07-04-2011	E 09-06-2011	F 24-05-2012
1. Patient activity VAS		62				
2. Physician activity VAS			42	57	9	42
3. MMT8	-	79	78	73	78	78
4. MMT24	-	256		233	246	250
5. HAQ	2.625	2.375	1.375	1.5	2.125	2.125
6. CK						
7. LDH						
8. MYOACT			0.05	0.03	0.01	0.03
9. *** MYOACT delta (%) ***						
10. MITAX		0.41	0.35	0.16	0.22	0.16
11. *** MITAX delta (%) ***						
12. Constitutional Disease Activity		11	22	10	0	5
13. Cutaneous Disease Activity		48	5	5	0	0
14. Skeletal Disease Activity		10	4	5	0	0
15. Gastrointestinal Disease Activity		8	0	0	0	9
16. Pulmonary Disease Activity		5	0	0	3	6
17. Cardiovascular Disease Activity			0	0	0	0
18. Muscle Disease Activity		9	5	39	5	21
19. Extramuscular Global Assessment		56	25	16	3	16
20. MDI Extent		0.12	0.22	0.16	0.14	0.12
21. MDI Severity		0.04	0.07	0.09	0.07	0.05
22. MDI Extended		0.13	0.07	0.2	0.13	0.07
23. ESR						
24. CRP						
25. Creatinine					54	
26. DLCO/VA pred.				82		
27. Improvement/Worsening status	N/A	-	-	-	-	-
28. outDLQI						
29. outEUROQOLFiveValue	-	-	-	-	-	-

Supplementary Appendix Figure 3: A sample scoreboard for a patient with DM. Serial clinical outcome measures are summarised.

Outputs

The benefits of a collaborative approach to rare disease research are increasingly being realised. For example, the largest genetic study in IIM was recently completed, largely using the EuroMyositis registry for case ascertainment and collection of clinical details [3]. In this study, the Human Leucocyte Antigen (HLA) region and *PTPN22* gene reached genome wide significance and distinct differences between disease subgroups were identified. Further work is ongoing, in particular a subgroup analysis focussing on patients with IBM and interactions between HLA alleles and serotype [4]. This study built on previous work using the precursors to

EuroMyositis in the UK (AOMIC and UKMYONET), where a number of important genetic [5,6] and serological [7] IIM associations were demonstrated, often in collaboration with others. In Mexico, analysis of patient data held within the EuroMyositis registry showed that possession of the *ACTN3* 577X allele appeared to increase the risk of developing IIM [8].

A number of projects have focussed on the elucidation of serological associations with IIM subtypes and certain clinical features. This includes recent work examining the rate of multiple MSA positivity in IIM [9] and a collaborative effort between the UK, The Netherlands, France and Sweden which has analysed the clinical implications of anti-CN-1a autoantibody positivity in patients with IBM [10]. In Mexico, the EuroMyositis registry has been used to characterise patients with IIM that have anti-Mi2 or anti-TIF1-gamma autoantibodies [11]. Data from the same group contributed to research regarding the implications of anti-TIF1-beta autoantibodies in those with DM [12]. Other ongoing projects include an analysis of factors predicting disease progression in patients with PM and DM which is being coordinated from Switzerland and an analysis of long term outcomes in childhood myositis which is being coordinated from the UK. This is already being implemented in the UK, where registry data is linked to nationally orchestrated morbidity and mortality data held by NHS Digital (formerly the Health and Social Care Information Service). This has facilitated investigations of the link between serological profile and mortality in cancer and non-cancer associated IIM [13].

Future developments

The EuroMyositis registry is currently undergoing modification to support the introduction of Fast Health Interoperability Resources (FHIR). FHIR is an emerging IT standard which facilitates exchange of health data by using agreed methods of describing data in different systems. This permits easier interoperability of IT systems and will support more widespread integration of the EuroMyositis registry with local electronic health records, without the requirement for bespoke synchronisation solutions. As such, the collection of routinely obtained data associated with the management of patients with IIM can automatically populate the registry avoiding the need for duplication and manual data input between different systems. Furthermore, linking to external systems provides a powerful method of investigating links between IIM and other factors such as the occurrence of comorbidities (including cancer) and mortality.

The EuroMyositis registry and underlying data storage platform is also integral to the UK Medical Research Council funded 'Prospective Cohort Study in Myositis' (MYOPROSP - https://clinicaltrials.gov/ct2/show/NCT02468895) and the Swedish quality of care register (SRO). Both studies are collecting standardised longitudinal data regarding national inception

cohorts of patients with IIM. In the future there will also be an increasing emphasis on collection of longitudinal patient reported outcome measures (PROMs). These data can be input directly in to the registry by the patient using a smartphone 'App' or other device (including 'wearable tech'). In addition, the Registry is being updated to employ more recently defined diagnostic/classification criteria and to harmonise data collection between the EuroMyositis Registry and other IIM registries.

Supplementary Appendix Table 1: Contributions to the EuroMyositis Registry per country.

**Diagnosis** *n (% within country)*	**Belgium**	**China**	**Czech Republic**	**Hungary**	**Italy**	**Mexico**	**Norway**	**Sweden**	**Switzerland**	**United Kingdom**	**Vietnam**	**Total** *n (%)*
Dermatomyositis	3 (7)	48 (46)	158 (40)	126 (28)	30 (38)	58 (62)	26 (29)	87 (24)	20 (39)	334 (27)	59 (39)	949 (31)
Polymyositis	2 (5)	20 (19)	75 (19)	273 (60)	30 (38)	23 (25)	12 (13)	57 (16)	9 (18)	220 (18)	92 (61)	813 (27)
Anti-synthetase Syndrome	2 (5)	15 (14)	74 (19)	21 (5)	7 (9)	5 (5)	34 (37)	73 (20)	15 (29)	266 (21)	0 (0)	512 (17)
Connective tissue disease-overlap myositis	6 (14)	11 (11)	45 (12)	12 (3)	10 (13)	0 (0)	10 (11)	67 (19)	2 (4)	195 (16)	0 (0)	358 (12)*
Inclusion Body Myositis	11 (25)	0 (0)	7 (2)	0 (0)	0 (0)	0 (0)	2 (2)	51 (14)	1 (2)	168 (14)	0 (0)	240 (8)
Immune-Mediated Necrotising Myopathy	20 (46)	2 (2)	23 (6)	3 (1)	1 (1)	2 (2)	3 (3)	5 (1)	3 (6)	43 (4)	0 (0)	105 (3)
Juvenile Dermatomyositis	0 (0)	8 (8)	11 (3)	21 (5)	2 (3)	6 (6)	4 (4)	18 (5)	1 (2)	19 (2)	0 (0)	90 (3)
Single visit – n (%)	44 (100)	104 (100)	137 (35)	443 (97)	44 (55)	94 (100)	88 (97)	163 (46)	16 (31)	1187 (95)	151 (100)	2471 (81)
2-5 visits – n (%)	0 (0)	0 (0)	151 (38)	13 (3)	26 (33)	0 (0)	3 (3)	58 (16)	35 (69)	55 (4)	0 (0)	341 (11)
6-10 visits – n (%)	0 (0)	0 (0)	75 (19)	0 (0)	10 (13)	0 (0)	0 (0)	72 (20)	0 (0)	3 (0)	0 (0)	160 (5)
>10 visits – n (%)	0 (0)	0 (0)	30 (8)	0 (0)	0 (0)	0 (0)	0 (0)	65 (18)	0 (0)	0 (0)	0 (0)	95 (3)
Median duration of follow up (in years) for those with >1 visit – (IQR), n	-	-	3.7 (1.8-6.5), 256	0.1 (0-0.2), 13	1.7 (1.2-2.0), 36	-	1.1 (1.1-1.2), 3	4.8 (2.4-7.9), 195	1.5 (0.9-2.1), 35	2.2 (1.3-4.1), 58	-	3.0 (1.5-6.1), 596
**Total** *n (% of total from all countries)*	44 (1)	104 (3)	393 (13)	456 (15)	80 (3)	94 (3)	91 (3)	358 (12)	51 (2)	1245 (41)	151 (5)	3067 (100)

Note that some countries have more than one centre.
*Associated connective tissue disease (CTD): Systemic sclerosis (39%, 141/358), Sjögrens syndrome (15%, 54/358), Mixed connective tissue disease (15%, 52/358), Rheumatoid arthritis (9%, 32/358), Systemic lupus erythematosus (9%, 32/358), Other (13%, 47/358).

Supplementary Appendix B

EuroMyositis Registry Definitions

Diagnostic criteria

PM,DM and JDM

Patients with PM, DM or JDM met the Bohan and Peter probable or definite diagnostic criteria [14,15]:

1. Symmetrical proximal muscle weakness.

2. Elevation of the serum enzymes, especially creatine phosphokinase, aldolase, aspartate aminotransferase, alanine aminotransferase and lactate dehydrogenase.

3. Abnormal electromyogram with myopathic motor unit potentials, fibrillations, positive sharp waves, increased insertional irritability and spontaneous bizarre high frequency discharges.

4. Muscle biopsy evidence of necrosis, phagocytosis, regeneration, degeneration, perifascicular atrophy, and an interstitial inflammatory infiltrate.

5. Typical skin rash of DM.

Definite polymyositis: criteria 1-4, probable polymyositis 3 of criteria 1-4

Definite dermatomyositis: criterion 5 and 3 of criteria 1-4, probable dermatomyositis criterion 5 and 2 of criteria 1-4.

Exclusion criteria for PM, DM and JDM

The EuroMyositis Registry includes the following additional condition regarding these criteria:

> ***The application of these criteria assumes that known infectious, toxic, metabolic, dystrophic, or endocrine myopathies have been excluded by appropriate evaluations***.

and specifically lists the following exclusion criteria:

1. Evidence of central or peripheral neurologic disease, including motor-neuron disorders with fasciculations or long-tract signs, sensory changes, decreased nerve conduction times, and fiber-type atrophy and grouping on muscle biopsy.

2. Muscle weakness with a slowly progressive, unremitting course and positive family history or calf enlargement to suggest muscular dystrophy.
3. Biopsy evidence of granulomatous myositis such as with sarcoidosis.
4. Infections, including trichinosis, schistosomiasis, trypanosomiasis, staphylococcosis and toxoplasmosis.
5. Recent use of various drugs and toxins, such as clofibrate and alcohol.
6. Rhabdomyolysis as manifested by gross myoglobinuria related to strenuous exercise, infections, crush injuries, occlusion of major limb arteries, prolonged coma or convulsions, high voltage accidents, heat stroke, the malignant-hyperpyrexia syndrome, and envenomation by certain sea snakes.
7. Metabolic disorders such as McArdle's syndrome.
8. Endocrinopathies such as thyrotoxicosis, myxedema, hyperparathyroidism, hypoparathyroidism, diabetes mellitus, or Cushing's syndrome.
9. Myasthenia gravis with response to cholinergics, sensitivity to d-tubocurarine, and decremental response to repetitive nerve stimulation.
10. Myositis after influenza and rubella infections, vaccination for rubella, the use of penicillamine, multicentric reticulohistiocytosis, giant-cell myositis, atheromatous microemboli, and carinomatous thromboembolization with muscle necrosis.

Anti-synthetase syndrome

Cases in the EuroMyositis Registry can be assigned a diagnosis of anti-synthetase syndrome (ASS) by the recruiting clinician if:

> ***"Clinical features of anti-synthetase syndrome are present together with an anti-synthetase antibody. Clinical features of anti-synthetase syndrome are: myositis, arthritis, interstitial lung disease, mechanic's hands, Raynaud's phenomenon, fever. The minimum for entering "yes" is the presence of anti-synthetase antibody and one clinical feature."***

It should be noted that this definition is consistent with the ASS classification criteria proposed by Conners *et al* that we applied to cases with PM, DM, JDM or CTD-overlap myositis. We undertook retrospective re-classification due to the fact that until recently, the results of several anti-synthetase autoantibody (ASA) results (particularly for the more novel ASAs) may not have been available to the recruiting clinician at the time of including a case in the EuroMyositis Registry.

Inclusion body myositis

IBM cases must meet either the Medical Research Council, Griggs *et al*, or European Neuromuscular Centre (ENMC) diagnostic criteria.[16–18]

Recent analysis of the competing IBM diagnostic criteria has suggested that whilst the differing criteria have wide-ranging sensitivities, the specificities are uniformly high.[19] Thus, we allowed the use of these different diagnostic criteria for IBM in our analysis to help improve sensitivity.

Connective tissue disease overlap myositis

The following diagnostic criteria were used for defining connective tissue diseases:

1. Systemic lupus erythematosus: Hochberg MC.[20]
2. Systemic sclerosis: American Rheumatism Association.[21]
3. Rheumatoid arthritis: Arnett *et al*.[22]
4. Sjögren's syndrome: Vitali C *et al.*[23]
5. Mixed connective tissue disease: Alarcón-Segovia D.[24]

Immune mediated necrotising myositis

Patients otherwise meeting the criteria for PM but with necrotic muscle fibres (in the absence of florid inflammation) as the predominant histological feature.

Clinically amyopathic-DM (CADM)

CADM is defined as a rare group of the IIM spectrum, in patients who have classic cutaneous findings confirmed by skin biopsy, in the absence of clinical, laboratory, or histologic evidence of myositis after at least 2 years of follow up.[25]

Other features

Cancer

Any malignancy regardless of whether considered as being "myositis associated" is recorded. Local practices for malignancy screening will vary from centre to centre.

Malignancy data presented in the attached manuscript is accurate up to July 2017.

Cardiac involvement related to the IIM disease process:

- Pericarditis: Inflammation of the pericardium defined clinically or by electrocardiogram (ECG) or echocardiogram
- Myocarditis: Inflammation of the myocardium defined clinically or with echocardiographic or other objective evidence
- Arrhythmia: clinical or ECG evidence of irregular heart beat

- Sinus tachycardia: resting heart rate > 100 beats per minute in an adult patient or greater than upper limit of age-appropriate normal value in a pediatric patient

Myositis-associated interstitial lung disease

- Radiologic (chest x-ray or chest CT scan) documentation of inflammation or scarring (fibrosis) of the parenchyma of the lung, and
- Abnormal PFT attributable to inflammatory process or pulmonary fibrosis.

Antibody status

The technique used for testing of autoantibodies was dependent upon local practice. The EuroMyositis Registry includes the following data regarding the methods used for determination of antibody status: Of the 1,951 cases with any antibody data available, 1,412 (72%) were determined by immunoprecipitation, 159 (8%) by ELISA and 84 (4%) by line blot. For 296 (15%) cases, the method for determining antibody status is not known.

Summary of Case Reclassification

We undertook a process of retrospective case reclassification to reflect the growing importance placed on serotype in determining classification. Patients recruited to the Registry prior to these developments may have been misclassified according to our current understanding. No new antibody testing or biopsy evaluations were performed as part of this study, but we did make use of information that may not have been available to clinicians at the time of patient recruitment. Details are included below:

1
- Raw data regarding idiopathic inflammatory myopathy (IIM) cases downloaded. Cases with no recorded diagnosis excluded. Remaining cases included statin related myotoxicity (SRM 1-6), clinically amyopathic dermatomyositis (CADM), dermatomyositis (DM), inclusion body myositis (IBM), "unspecified myositis", juvenile dermatomyositis (JDM), juvenile polymyositis (JPM), polymyositis (PM) and "pure" anti-synthetase syndrome (i.e. no other IIM subtype selected).

2
- Cases of PM, DM, JDM or unspecified myositis with a co-existing connective tissue disease (CTD) meeting relevant diagnostic criteria* were reclassified as CTD-overlap myositis

3
- Any case meeting criteria for anti-synthetase syndrome (ASS)*, either alone ("pure" ASS) or in combination with another IIM subtype, according to recruiting clinician were reclassified as ASS

4
- Cases originally classified as PM, DM, CADM, JDM and cases assigned to the CTD-overlap myositis category were reclassified as ASS if they met criteria suggested by Connors *et al.* * / **
- Remaining cases with ADM were reclassified as DM

5
- Cases with SRM6 re-classified as immune mediated necrotising myopathy (IMNM). Remaining cases of SRM (SRM1-5), and cases of PM, where the patient also had anti-HMGCR autoantibodies were reclassified as IMNM
- Cases of PM with anti-SRP or anti-HMGCR autoantibodies reclassified as IMNM

6
- Remaining cases of SRM1-5, unspecified myositis, or JPM were removed from the analysis

7
- Cases of DM & JDM excluded where confirmation could not be found that a DM-associated rash was present
- Cases of PM, IBM, IMNM excluded if presence of DM-associated rash recorded

*See EuroMyositis Registry Definitions section of Supplementary Appendix B for full details of definitions. **Note that this did not include any patients from the CTD-overlap myositis group that were originally classified as "unspecified myositis".

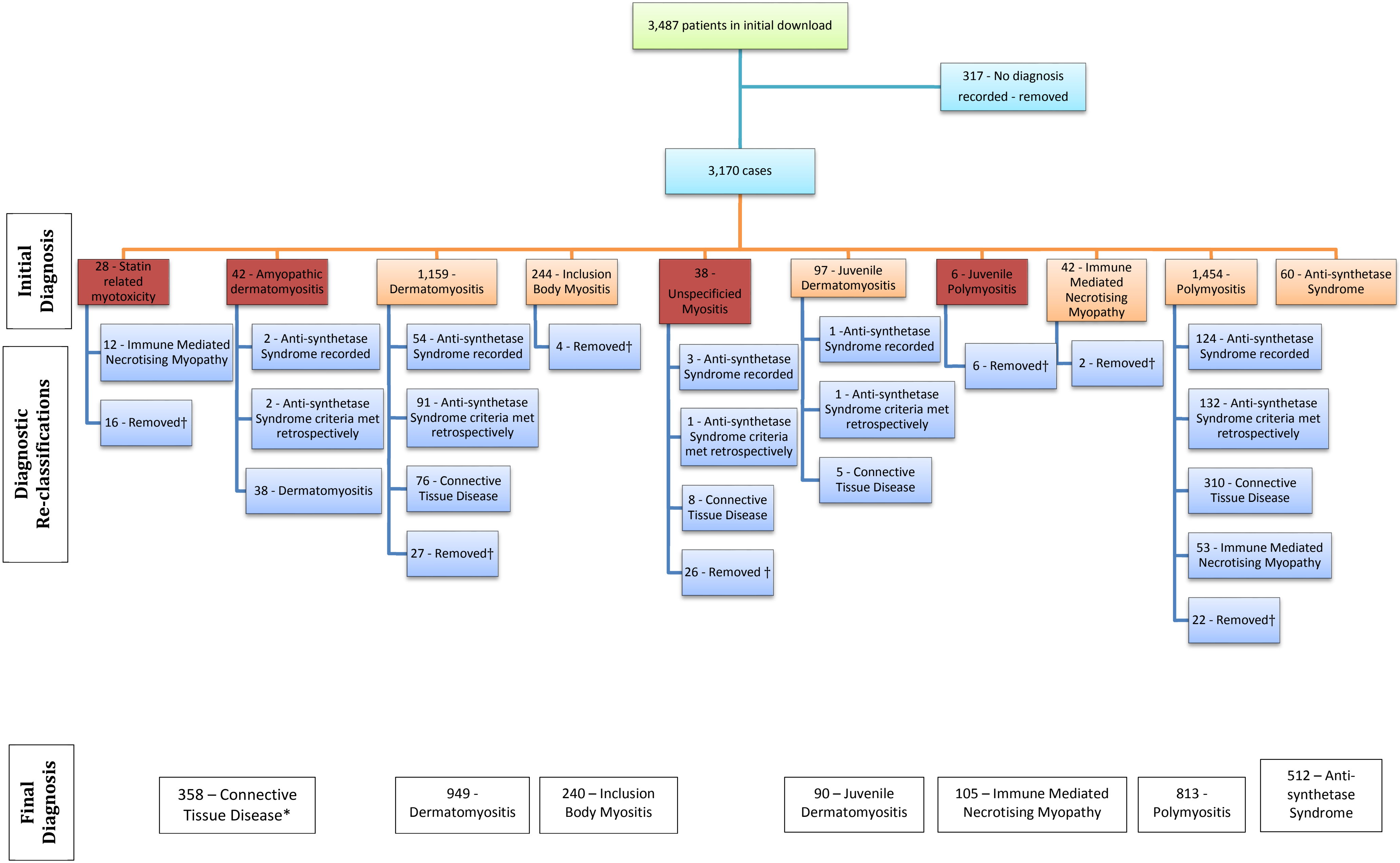

*Excludes 41 patients retrospectively reclassified as Anti-synthetase Syndrome and analysed as ASS, rather than with the CTD-overlap group. Subtype classifications are mutually exclusive. “Anti-synthetase Syndrome Recorded” implies that the presence of Anti-synthetase Syndrome was already flagged in the Registry against cases with other IIM subtypes. “Anti-synthetase Syndrome criteria met retrospectively” implies that we were able to reclassify the case as Anti-synthetase Syndrome using criteria proposed by Connors *et al* with the information available in the Registry.
† See accompanying explanations for exclusion reasons

References

1 Isenberg DA, Allen E, Farewell V, *et al.* International consensus outcome measures for patients with idiopathic inflammatory myopathies. Development and initial validation of myositis activity and damage indices in patients with adult onset disease. *Rheumatology* 2004;43:49–54.
2 Miller FW. New approaches to the assessment and treatment of the idiopathic inflammatory myopathies. *Ann Rheum Dis* 2012;71 Suppl 2:i82-5.
3 Rothwell S, Cooper RG, Lundberg IE, *et al.* Dense genotyping of immune-related loci in idiopathic inflammatory myopathies confirms HLA alleles as the strongest genetic risk factor and suggests different genetic background for major clinical subgroups. *Ann Rheum Dis* 2016;75:1558–66.
4 Rothwell S, Cooper RG, Lundberg IE, *et al.* Immune-array Analysis in Sporadic Inclusion Body Myositis Reveals HLA-DRB1 Amino Acid Heterogeneity across the Myositis Spectrum. *Arthritis Rheumatol* Published Online First: 13 January 2017.
5 Miller FW, Chen W, O'Hanlon TP, *et al.* Genome-wide association study identifies HLA 8.1 ancestral haplotype alleles as major genetic risk factors for myositis phenotypes. *Genes Immun* 2015;16:470–80.
6 Chinoy H, Adimulam S, Marriage F, *et al.* Interaction of HLA-DRB1*03 and smoking for the development of anti-Jo-1 antibodies in adult idiopathic inflammatory myopathies: a European-wide case study. *Ann Rheum Dis* 2012;71:961–5.
7 Tansley SL, Betteridge ZE, Gunawardena H, *et al.* Anti-MDA5 autoantibodies in juvenile dermatomyositis identify a distinct clinical phenotype: a prospective cohort study. *Arthritis Res Ther* 2014;16:R138.
8 Sandoval-García F, Petri M, Saavedra M, *et al.* The ACTN3 R577X polymorphism is associated with inflammatory myopathies in a Mexican population. *Scand J Rheumatol* 2012;41:396–400.
9 Betteridge ZE, Chinoy H, Cooper RG, *et al.* 175 Myositis-Specific Autoantibodies Rarely Coexist with Each Other: An Analysis of the Ukmyonet and Eumyonet Cohorts. *Rheumatology* 2016;55:i133–i133.
10 Lilleker JB, Rietveld A, Pye SR, *et al.* Cytosolic 5'-nucleotidase 1A autoantibody profile and clinical characteristics in inclusion body myositis. *Ann Rheum Dis* 2017;76:862–8.
11 Petri MH, Satoh M, Martin-Marquez BT, *et al.* Implications in the difference of anti-Mi-2 and -p155/140 autoantibody prevalence in two dermatomyositis cohorts from Mexico City and Guadalajara. *Arthritis Res Ther* 2013;15:R48.
12 Satoh M, Chan JYF, Ross SJ, *et al.* Autoantibodies to transcription intermediary factor TIF1β associated with dermatomyositis. *Arthritis Res Ther* 2012;14:R79.
13 Oldroyd AGS, Newton LR, Sergeant JC, *et al.* The Risk of Premature Death of Both Cancer-Associated and Non-Cancer–Associated Mysositis in UK Adult-Onset Myositis Patients is Significantly Increased Compared with the General Population. *Rheumatology* 2016;55:i46.
14 Bohan A, Peter JB. Polymyositis and Dermatomyositis. *N Engl J Med* 1975;292:344–7.
15 Bohan A, Peter JB. Polymyositis and dermatomyositis (second of two parts). *N Engl J Med* 1975;292:403–7.
16 Hilton-Jones D, Miller A, Parton M, *et al.* Inclusion body myositis: MRC Centre for

Neuromuscular Diseases, IBM workshop, London, 13 June 2008. *Neuromuscul Disord* 2010;20:142–7.
17 Griggs RC, Askanas V, DiMauro S, *et al.* Inclusion body myositis and myopathies. *Ann Neurol* 1995;38:705–13.
18 Verschuuren JJ, Badrising UA, Van Engelen BG, Van der Hoeven JH, Hoogendijk J WA. Inclusion body myositis. In: AEH E, ed. *Diagnostic Criteria for Neuromuscular Disorders*. London: : Royal Society of Medicine Press 1997. 81–4.
19 Lloyd TE, Mammen AL, Amato A a, *et al.* Evaluation and construction of diagnostic criteria for inclusion body myositis. *Neurology* 2014;83:426–33.
20 Hochberg MC. Updating the American College of Rheumatology revised criteria for the classification of systemic lupus erythematosus. *Arthritis Rheum* 1997;40:1725.
21 Preliminary criteria for the classification of systemic sclerosis (scleroderma). Subcommittee for scleroderma criteria of the American Rheumatism Association Diagnostic and Therapeutic Criteria Committee. *Arthritis Rheum* 1980;23:581–90.
22 Arnett FC, Edworthy SM, Bloch DA, *et al.* The American Rheumatism Association 1987 revised criteria for the classification of rheumatoid arthritis. *Arthritis Rheum* 1988;31:315–24.
23 Vitali C, Bombardieri S, Moutsopoulos HM, *et al.* Preliminary criteria for the classification of Sjögren’s syndrome. Results of a prospective concerted action supported by the European Community. *Arthritis Rheum* 1993;36:340–7.
24 Alarcón-Segovia D. Mixed connective tissue disease and overlap syndromes. *Clin Dermatol* 1994;12:309–16.
25 Santmyire-Rosenberger B, Dugan EM. Skin involvement in dermatomyositis. *Curr Opin Rheumatol* 2003;15:714–22.
